# Evaluation of Various Lactic Acid Bacteria and Generic *E. coli* as Potential Nonpathogenic Surrogates for In-Plant Validation of Biltong Dried Beef Processing

**DOI:** 10.3390/microorganisms10081648

**Published:** 2022-08-15

**Authors:** Caitlin E. Karolenko, Jade Wilkinson, Peter M. Muriana

**Affiliations:** 1 Robert M. Kerr Food and Agricultural Product Center, Oklahoma State University, Stillwater, OK 74078, USA; 2 Department of Animal and Food Sciences, Oklahoma State University, Stillwater, OK 74078, USA

**Keywords:** biltong, surrogate, lactic acid bacteria, dried beef, validation, *Carnobacterium*

## Abstract

Validation studies conducted within a food processing facility using surrogate organisms could better represent the manufacturing process than controlled laboratory studies with pathogenic bacteria on precision equipment in a BSL-2 lab. The objectives of this project were to examine potential surrogate bacteria during biltong processing, conduct biltong surrogate validation lethality studies, and measure critical factors and intrinsic parameters during processing. Beef pieces (1.9 cm × 5.1 cm × 7.6 cm) were inoculated with four-strain mixtures of *Carnobacterium* *divergens/C. gallinarum*, *Pediococcus acidilactici/P. pentosaceous*, and Biotype 1 *E. coli* ATCC BAA (-1427, -1428, -1429, and -1430), as well as a two-strain mixture of *Latilactobacillus sakei* and other commercially available individual bacterial cultures (*P. acidilactici* Saga200/Kerry Foods; *Enterococcus faecium* 201224-016/Vivolac Cultures). Inoculated beef was vacuum-tumbled in marinade and dried in a humidity-controlled oven for 8–10 days (24.9 °C; 55% relative humidity). Microbial enumeration of surviving surrogate bacteria and evaluation of intrinsic factors (water activity, pH, and salt concentration) were performed post inoculation, post marination, and after 2, 4, 6, 8, and 10 days of drying. Trials were performed in duplicate replication with triplicate samples per sampling time and analyzed by one-way RM-ANOVA. Trials conducted with *E. faecium*, *Pediococcus* spp., and *L. sakei* never demonstrated more than 2 log reduction during the biltong process. However, *Carnobacterium* achieved a >5 log (5.85 log) reduction over a drying period of 8 days and aligned with the reductions observed in previous trials with pathogenic bacteria (*Salmonella, E. coli* O157:H7, *L. monocytogenes*, and *S. aureus*) in biltong validation studies. Studies comparing resuspended freeze-dried or frozen cells vs. freshly grown cells for beef inoculation showed no significant differences during biltong processing. *Carnobacterium* spp. would be an effective nonpathogenic in-plant surrogate to monitor microbial safety that mimics the response of pathogenic bacteria to validate biltong processing within a manufacturer’s own facility.

## 1. Introduction

Biltong is a South African style dried beef product that is growing in popularity in the United States. This dried meat product is traditionally made using lean strips of beef that are marinated in a mixture of traditional spices (coriander and pepper), salt, and vinegar and then dried at low or ambient temperature and humidity. Dried beef processing guidelines, as issued by the United States Department of Agriculture Food Safety and Inspection Service (USDA-FSIS), require dried beef products to be heated to an internal temperature of 160 °F (71.1 °C) in a sealed oven or steam injector with a relative humidity greater than 90% during the cooking/heating process [1]. Since biltong does not have a heat lethality step during processing and deviates from these guidelines, biltong manufacturers must conduct a validation or challenge study to evaluate the ability of their process to sufficiently inactive bacterial pathogens such as *Salmonella* spp. which have been historically linked to outbreaks and recalls of dried meat and poultry products [2]. USDA-FSIS does give processors two different options to safely produce these alternative dried meat products. The first option requires *Salmonella* testing of every lot of edible ingredients used during processing and an overall process reduction of a ‘pathogen of concern’ of at least 2 log. Alternatively, processors can forego ingredient testing if they can demonstrate that their process can achieve ≥ 5 log reduction of *Salmonella* by the end of processing [3].

USDA-FSIS regulatory guidance for manufacture and sale of biltong requires processors to demonstrate product safety by process validation against a ‘pathogen of concern’. In recent BSL-2 in-lab studies, this was performed with *Salmonella* serovars [4], *E. coli* O157:H7, *L. monocytogenes*, and *S. aureus* [5]. These experiments, while successful in achieving a >5 log reduction of foodborne pathogens where the data are currently used by processors in support of their in-plant food safety (HACCP) processes, are often conducted in highly controlled BSL-2 laboratory environments with research-grade equipment. The food processing environment is extremely variable between small and large processors, and both likely have greater variability of process parameters than that found in BSL-2 lab equipment. USDA-FSIS has recognized this difference and has allowed consideration of ‘in-plant’ validation studies using surrogate organisms if the surrogate can mimic a pathogen’s response to a process [6,7,8]. The intention is that in-plant data would more likely reflect the actual process variability and conditions than scientific equipment from a BSL-2 lab. Conducting a validation study within a processor’s own facility would allow for a more accurate representation of the impact of a commercial process on pathogenic bacteria. Due to food safety concerns, it is unsafe to introduce pathogenic bacteria into a manufacturing facility to test whether the process achieves sufficient microbial reduction. Therefore, nonpathogenic surrogate bacteria would be better suited to mimic the response of pathogens to actual processing conditions [8]. This presents the following question: what surrogate organism should be used for the biltong process?

A surrogate organism for a challenge study is typically a nonpathogenic organism that has similar survival capabilities and susceptibility to injury as the target pathogen and closely mimics how the pathogen would react under similar processing conditions [9,10]. A variety of organisms have been used as surrogates in place of pathogens to mimic pathogenic responses in commercial food processes, predominantly *E. faecium*, *Pediococcus* spp., and Biotype 1 *E. coli*. *Enterococcus faecium* ATCC 8459 (NRRL B-2354), used as a surrogate for *Salmonella* Enteritidis PT 30 in the thermal processing of wheat flour [11], as a *S. enterica* surrogate for storage time and temperature of milk powders [12], in thermal extrusion of low-moisture foods [13], and in plant-level validation of thermal processes for peanuts and pecans [14]. Investigators also found that *Pediococcus* strains had similar heat tolerances to *Salmonella* spp. and would be suitable surrogates for validation studies of jerky-style dried meat products [15,16,17]. *Pediococcus acidilactici* ATCC 8042 was examined as a *Salmonella* surrogate for thermal processing of toasted oats for cereal and peanuts for peanut butter [18], and for processing of low-moisture pet food [19]. Biotype 1 *E. coli* ATCC BAA-1427, BAA-1428, BAA-1429, and BAA-1430 have been used as thermal surrogates for *E. coli* O157:H7 in meat processes [20], as *Salmonella* surrogates for thermal processing of ground beef [21], and for thermal treatment of almonds and pistachios [22,23]. These strains have been recommended by USDA-FSIS as surrogate indicator organisms for food process validation studies [8].

Despite the prevalence of studies performed with surrogate bacteria for various food processes, no surrogate organisms have been proven to suitably represent the response of pathogens during biltong processing. The objective of this study was to examine potential nonpathogenic lactic acid bacteria and generic *E. coli* strains that could be used for in-plant studies to mimic pathogen lethality during biltong processing.

## 2. Materials and Methods

### 2.1. Bacterial Strains and Growth Conditions

Bacterial cultures used in this study were obtained from various sources including our laboratory culture collection, commercial starter cultures, and bacteria isolated from biltong trials as listed in Table 1.

Bacterial isolates obtained from previous biltong beef trials after marination and drying for 8 days at 24.9 °C (75 °F) and 55% relative humidity (RH) were identified by 16S rRNA PCR/sequencing [24] as *Carnobacterium gallinarum*, *Carnobacterium divergens*, and *Latilactobacillus sakei* for examination as biltong process surrogates (Table 1).

Other lactic acid bacteria used in this study included *Pedicoccus acidilatcici* ATCC 8042, *P. acidilactici* P02K5, *P. pentosaceus* FBB61-2, and *P. pentosaceus* ATCC 43200, which are maintained in our laboratory culture collection. Some of these strains have been evaluated in other surrogate studies [19,25]. Nonpathogenic *E. coli* ATCC BAA-1427, BAA-1428, BAA-1429, and BAA-1430 have been used as Biotype 1 surrogate strains in various process validation studies and recommended for such use by USDA-FSIS [8,20,26]. *P. acidilactici* Saga200, used as a protective starter culture, was obtained as a frozen slurry from Kerry Foods (Beloit, WI, USA). *Enterococcus faecium* 201224-016 was obtained as a freeze-dried powder from Vivolac Cultures (Indianapolis, IN, USA) and is sold as a probiotic.

*Carnobacterium* spp., *E. faecium*, and *E. coli* cultures were inoculated into tryptic soy broth (TSB, BD Bacto, Franklin Laes, NJ, USA) and grown at 30 °C for 24 h. *L. sakei* and *Pedicoccus* spp. were inoculated into De Man, Rogosa and Sharpe broth (MRS, BD Bacto) and grown at 30 °C for 24 h. Cultures were prepared for storage by centrifugation (7200× *g*, 5 °C) of 9 mL of fresh, overnight culture, and the resulting pellet was resuspended with 2–3 mL of fresh, sterile TSB or MRS broth containing 10% glycerol. The cells in freezing media were then placed in 8 mL sterile glass vials and stored in an ultralow-temperature freezer (−80 °C) until use. Prior to use, frozen stocks were revived by transferring 100 µL of partially thawed culture into 9 mL of either TSB or MRS broth and incubated overnight at 30 °C.

Several cultures were used directly after suspension from the freeze-dried or frozen state for comparison of biltong process performance with metabolically active forms grown in liquid media. Prior to use, *P. acidilactici* Saga200 (frozen) was resuspended by adding 0.5 g of the frozen culture to 9 mL of 0.1% buffered peptone water (BPW, BD Difco) and vortexing until completely incorporated. *E. faecium* 201224-016 was resuspended by adding 0.1 g of the freeze-dried culture to 9 mL of 0.1% BPW and vortexing until completely mixed.

### 2.2. Acid Adpation of Cultures

Acid adaptation of active four-strain mixtures of *Carnobacterium* spp., *Pediococcus* spp., and *E. coli* BAA-strains was conducted as first described by Wilde et al. [27] and as used in previous biltong studies [4,28]. In brief, individual cultures were inoculated into TSB or MRS containing 1% glucose, incubated overnight at 30 °C, and harvested by centrifugation; cell pellets were then resuspended with 0.1% BPW. For mixed culture biltong inocula, individual strains were cultured, centrifuged, resuspended, and then combined in equal proportions to create a mixed inoculum cocktail. The commercial starter cultures (*P. acidilactici* Saga200 and *E. faecium* 201224-016) were not acid-adapted and used as a single-strain inoculum.

### 2.3. Beef Sample Preparation and Inoculation

USDA select-grade boneless beef rounds were obtained from a local meat processor (Ralph’s Perkins, OK, USA) who obtains beef from a wholesale beef broker. Beef rounds were trimmed of fat and cut into approximately 5.1 cm wide × 1.9 cm thick × 7.6 cm long beef squares and held overnight at 5 °C on foil-lined trays wrapped in plastic bags. Beef pieces were inoculated the following morning with the respective inoculum depending on the trials being performed that day. Beef pieces were inoculated with the *Carnobacterium* spp. mixture (*C. divergens* GO-R2E-B, GO-R1B; *C. gallinarum* NB-R2A, NB-R2B), the *L. sakei* mixture (*L. sakei* GO-R2C, GO-R2D), the *Pediococcus* spp. mixture (*P. acidilactici* ATCC 8042, PO2K5; *P. pentosaceous* ATCC 43200, FBB61-2), *P. acidilactici* Saga200, or *E. faecium* 201224-016. The inoculum suspension (150 µL) was applied to each side of the beef pieces and immediately spread with a gloved finger. Inoculated beef pieces were then allowed to incubate for 30 min at 5 °C to allow for bacterial attachment prior to use.

### 2.4. Biltong Processing, Marination, and Drying

Biltong processing was conducted as described whereby trials were performed in duplicate and triplicate samples were harvested at each timepoint (*n* = 6) [4,29]. Following inoculation and attachment, the beef pieces were then dipped in sterile water to mimic rinse treatments that processors often apply using antimicrobials or water during processing. The inoculated pieces were placed in a plastic basket, dipped in sterile water in a stainless-steel tub for 30 s, and drained for 60 s to release excess liquid. The beef pieces were then placed into a chilled metal tumbling vessel containing a biltong marinade. The biltong marinade consisted of 2.2% salt, 0.8% black pepper, 1.1% coarse ground coriander, and 4% red wine vinegar (100-grain; 10% acetic acid) in relation to the total meat weight. Beef pieces were vacuum-tumbled (15 inches Hg) in a Biro VTS-43 vacuum-tumbler (Marblehead, OH, USA) for 30 min and then hung to dry in a humidity-controlled oven (Hotpack, Model 435315, Warminster, PA, USA) at 55% relative humidity and 24.9 °C (75 °F) for 8–10 days.

### 2.5. Selective Recovery of Inoculum Bacteria from Biltong-Inoculated Beef

The bacteria assessed in this study as potential biltong processing surrogates were inoculated onto raw beef, and initial and residual inoculum enumeration had to preclude other natural contaminants also found on raw beef, those contributed during trimming of beef, or from the marinade spice mix. Prior studies indicated that such processing conditions induce stresses, and injured cells may not be recovered on harsh selective media, thereby giving a falsely lower count [28]. To eliminate the possibility of inhibiting injured-but-viable cells, we used generic growth media (TSA, MRS agar) supplemented with antibiotics to which the strains are resistant as a selective medium to enumerate our inoculated organisms from samples taken during biltong processing [4,28]. Antibiotic resistance was determined using antibiotic susceptibility discs (BD BBL Sensi-Discs, BD Labs, Franklin Lakes, NJ, USA) to determine innate antibiotic resistance (Table 1). After identification of antibiotic resistances, cultures were then enumerated on media with and without antibiotics to ensure the absence of inhibition from the use of antibiotics in the media as described previously [4,28,30]. For some strains used as inoculum cocktails that did not have consensus of the same antibiotic resistances, antibiotic resistance was acquired by plating on low level antibiotics known to generate spontaneous antibiotic resistance (i.e., gentamycin and rifamycin).

### 2.6. Comparison of Commerically Available Starter Cultures as Biltong Inoculants in their Lyophilized and Metabolically Active Forms

#### 2.6.1. Culture Preparation

Lactic acid bacteria obtained as freeze-dried cultures from starter culture companies for use in validation studies may present a facile method of use as validation inocula by simply resuspending the cells in buffer and directly inoculating beef samples [15,17]. Freeze-drying or lyophilization of bacteria exposes them to stressful conditions that can affect subsequent cell viability or activity [20,21]. Therefore, the activity of lyophilized (*E. faecium* 201224-016) and frozen (*P. acidilactici* Saga200) starter cultures and their metabolically active forms (i.e., after growth in media) were compared in their response to biltong processing.

For the lyophilized culture (*E. faecium* 201224-016), 0.1 g of freeze-dried powder was added to 9 mL of sterile 0.1% BPW and vortexed until completed suspended. The resuspended mixture was then used to inoculate each beef piece (300 µL; 150 µL/side) prior to marination.

For the frozen starter culture (*P. acidilactici* Saga200), a sterile hollow hole puncher was used to core ~0.8 g of frozen Saga200 from the manufacture’s container which was added to 9 mL of sterile 0.1% BPW and vortexed until mixed. The culture suspension was kept chilled on ice and used shortly thereafter to inoculate beef pieces.

Metabolically active versions of these cultures were obtained by growth in 150 mL of the appropriate media (TSB, MRS) for 24 h at 30 °C, centrifugation, and resuspension of the recovered cell pellet with 5 mL of sterile 0.1% BPW. The resuspended culture was then used to inoculate beef pieces prior to use in the validation study. The lyophilized and metabolically active forms of *E. faecium* 201224-016 and *P. acidilactici* Saga200 were used in parallel and simultaneous biltong trials to reduce any variables that might influence the observed effect of the marinade and drying process.

#### 2.6.2. Lyophilization of *Carnobacterium gallinarum* NB-R2A

To the authors’ knowledge, there is no commercially available *Carnobacterium* strain available in the United States. Therefore, *C. gallinarum* NB-R2A, isolated from biltong, was lyophilized via freeze-drying to examine a lyophilized version for comparison with the actively grown culture. *Carnobacterium gallinarum* NB-R2A was inoculated into 9 mL of TSB from frozen stock and incubated for 18 h at 30 °C. Following incubation, the 9 mL culture was transferred to 190 mL of TSB and incubated again for 18 h at 30 °C. The culture was then centrifuged at 7200× *g* for 20 min. The supernatant was removed, and the cell pellet was resuspended with 5 mL of sterile BPW and repeated. The supernatant was removed following centrifugation, and the final cell pellet was resuspended with 10 mL of autoclaved milk-based freeze-drying medium consisting of 11 g of skim milk powder, 1 g of dextrose, 1 g of trehalose, and 0.2 g of yeast extract per 100 mL. The milk/cell suspension was added to Oak Ridge tubes (5 mL each) and freeze-dried using a Heto vacuum centrifuge (Model VR-maxi) connected to a Heto freezing condensor (Model CT 60E) and a Leybold Trivac vacuum pump (Model D2.5F) setup for 24 h under vacuum. The freeze-dried powder was then stored at −80 °C until use in our biltong study. Just before use, 0.25 g of powder was added to 9 mL of sterile 0.1% BPW, vortexed until mixed, and used to inoculate beef pieces for biltong processing.

### 2.7. Evaluation of Critical Parameters and Intrinsic Factors in the Biltong Process

#### 2.7.1. Water Activity

Uninoculated beef pieces were sampled for water activity (A_w_) measurements at various stages throughout processing (in triplicate) including the initial raw beef, beef after marination, and then beef after drying for 2, 4, 6, 8, and 10 days. To obtain measurements, beef pieces were cut in half and placed in a sampling cup with the interior portion of the sample facing upward (toward the sensor). Samples were then covered with sampling cup cover containing the sensor and allowed to equilibrate to the temperature of the room. Water activity was measured using a HC2-AW-USB probe with a direct PC interface and HW4-P-Quick software (Rotronic Corp., Hauppauge, NY, USA). Measurements were taken in triplicate for each sample at each timepoint.

#### 2.7.2. Moisture Loss

Following marination, each beef piece was individually weighed and labeled prior to being hung in the humidity-controlled oven. Three pieces were selected and weighed prior to processing, and then sampled every 2 days while drying. The weight at the time of sampling was compared to the initial weight of the same piece recorded prior to drying. The determination of percent moisture loss was calculated as per Equation (1).

(1) $$\%\;Moisure\;Loss=\frac{\left[\left(inital\;weight\right)-\left(final\;weight\right)\right]}{\left(inital\;weight\right)}\times 100$$.

#### 2.7.3. Measurement of Biltong Beef pH

Measurements of beef pH were obtained at various points in the biltong process including raw beef, beef following marination, and beef after 2, 4, 6, 8, and 10 days of drying. At each timepoint, three pieces of uninoculated beef were collected, weighed, and then added to a laboratory blender with steel blades (Waring Commercial, New Harford, CT, USA) with sterile water of equal weight to the weight of the beef pieces. The water and beef mixtures were blended until a finely ground mixture was formed. The pH of the homogenized meat mixture was measured in triplicate using an H-series pH meter and probe (Hach, Loveland, CO, USA).

#### 2.7.4. Salt Concentration

The homogenized meat mixture used to measure pH was also used to obtain salt concentrations of each sample. Horiba LAQUA Twin Pocket Meter (Horiba Instruments, Irvine, CA, USA) was used to quantify sodium ion concentration. Approximately 300 µL of the homogenized sample was placed in the sample chamber and allowed to stabilize before recording. Readings (in ppm) were taken in triplicate for each sample. To determine the salt (*NaCl*) concentration from the sodium ion concentration, the following equations were used:

(2) $$Na\;\left(\frac{\mathrm{mg}}{100\;g}\right)=Meter\;reading\;\left(\mathrm{ppm}\right)\times \frac{Weight\;after\;Dilution\;\left(g\right)}{Sample\;Weight}\times 100,$$

(3) $$NaCl\;Salt\;\left(\frac{g}{100\;g}\right)=Na\;\left(\frac{\mathrm{mg}}{100\;g}\right)\times \frac{NaCl\;molar\;mass}{Na\;molar\;mass}\times \frac{1}{1000}.$$

### 2.8. Microbial Sampling and Inoculum Enumeration of Biltong Beef

At each sampling timepoint of biltong beef processing (raw beef, after marinade, and after every 2, 4, 6, 8, and 10 days of drying), three beef pieces were selected at random and placed in a sterile Whirl-pak filter stomaching bag (Nasco, Fort Atkinson, WI, USA) in combination with 100 mL of 1% neutralizing buffered peptone water (nBPW, Criterion, Hardy Diagnostics, Santa Maria, CA, USA). Samples were stomached for 60 s in a paddle-blender masticator (IUL Instruments, Barcelona, Spain). Serial dilutions were made with 1% BPW and plated on TSA containing gentamicin and rifamycin (2.5 µg/mL each) for *Carnobacterium*, on MRSA containing gentamicin and rifamycin (2.5 µg/mL each) for *L. sakei*, on MRSA containing gentamicin (10 µg/mL) and rifamycin (5 µg/mL) for *Pediococcus* spp., on TSA containing naldixic acid and colistin (10 µg/mL each) for *E. faecium* 201224-016, or on MRS containing nalidixic acid and colistin (10 µg/mL each) for *P. acidilactici* Saga200; the filter bag dilution was considered the 10° dilution. Plates were incubated at 30 °C for 48 h and enumerated as log CFU/mL. Samples were collected in triplicate replication and plated in duplicate at each sampling timepoint.

### 2.9. Statistical Analysis

Validation trials were conducted in duplicate with triplicate sampling at each timepoint (*n* = 6) as per validation criteria established by the National Advisory Committee on Microbial Criteria for Foods (NACMCF) [9] and supported by the USDA-FSIS [31]. Data are presented as the mean of multiple replications with standard deviation of the mean represented by error bars. Statistical analysis of data collected over time was performed using one-way repeated-measures analysis of variance (RM-ANOVA). Pairwise multiple comparisons were performed using the Holm–Sidak test to determine significant differences. Data treatments with the same letter are not significantly different (*p* > 0.05); treatments with different letters are significantly different (*p* < 0.05).

## 3. Results and Discussion

### 3.1. Critical Parameters and Intrinsic Factors

#### 3.1.1. Water Activity, Moisture Loss, and Salt Concentrations

**Figure 1:**
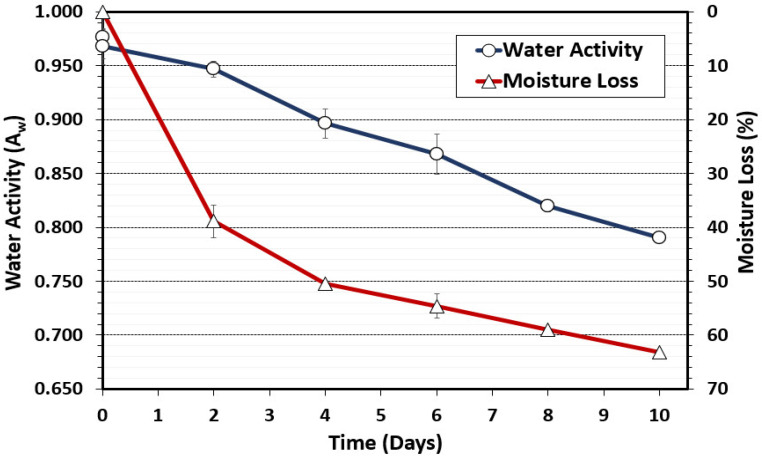
Water activity (A_w_) and moisture loss during biltong processing at 24.9 °C (75 °F) and 55% RH. The data represent the average of measurements taken during duplicate trials with triplicate samples taken at each time interval (*n* = 6).

To complement the surrogate validation trials, we measured and recorded critical operational parameters and intrinsic factors at each key stage of processing (raw beef, inoculation, marination, and every 2 days of drying) as recommended by USDA-FSIS [1]. Water activity (A_w_) is a measure of free, unbound water available for bacterial growth. USDA-FSIS considers vacuum-tumbled beef as ‘nonintact beef’, whereby A_w_ is a primary safety factor as there is no heat lethality step in biltong processing and biltong is processed as thick beef samples [32,33]. Therefore, A_w_ is a critical safety factor for control of bacteria that might be internalized due to vacuum tumbling. *S. aureus* that can tolerate low A_w_ and high salt levels would be a concern for possible production of staphylococcal enterotoxin. The targeted A_w_ for shelf-stable beef jerky is <0.85 which was achieved after 7 days of drying (Figure 1) [1,2]. Water activity after 8 and 10 days of drying ranged from 0.82 to 0.79 respectively. Similarly, beef samples showed incremental moisture loss with 59% and 62.5% loss at 8 and 10 days, respectively (Figure 1).

**Figure 2:**
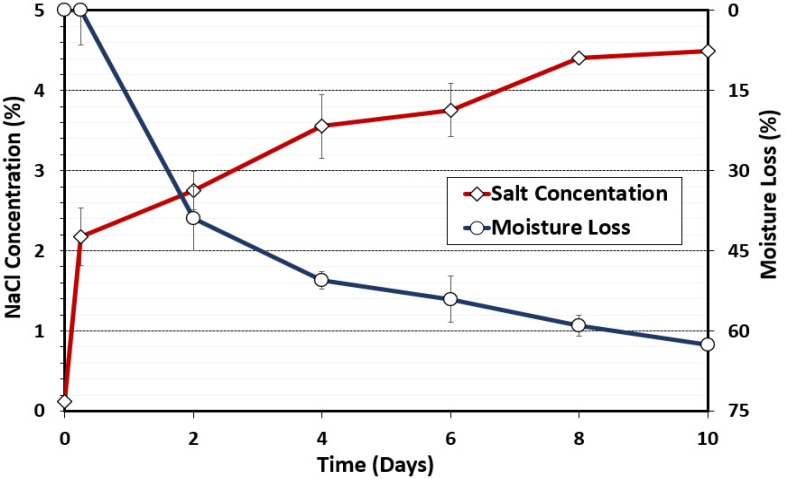
Moisture loss (%) and salt concentration (%) during biltong processing. Measurements were taken with initial beef samples, after marination, and after 2, 4, 6, 8, and 10 days of drying at 24.9 °C (75 °F) and 55% RH. Data points represent the mean of duplicate trials with triplicate samples taken at each time interval (*n* = 6).

Salt concentration was also determined during the biltong process. Salt concentration was calculated from sodium readings obtained with the LAQUAtwin NA-11 sodium ion meter (Horiba Inc, Irvine, CA, USA). The initial calculated salt concentration determined on raw beef was 0.12% NaCl; then, following the marination step, the beef salt concentration shot up to 2.17% (2.17 g NaCl/100 g beef). The initial salt level falls in line with expectations given that the biltong marinade was formulated at 2.2% salt (*w*/*w*). The salt concentration increased over time and was indirectly proportional to moisture loss during the drying process (Figure 2). As expected, as moisture loss occurred, A_w_ was also reduced to below 0.85 A_w_ (Figure 1) and the salt concentration increased to above 4% (Figure 2); both conditions are inhibitory to most bacteria, helping to ensure a safe product for consumers [34]. Biltong safety involves an interplay among moisture, salt concentration, and A_w_ since moisture loss increases salt concentration, while salt binds water and helps to draw it out of the interior of the beef, thereby reducing A_w_. For consumer issues regarding high sodium levels, the use of alternative salts (CaCl_2_, KCl) instead of NaCl can help lower sodium levels in finished biltong while still maintaining a 5 log reduction of pathogen (*Salmonella*) [29].

#### 3.1.2. The pH of Beef during Biltong Processing

**Figure 3:**
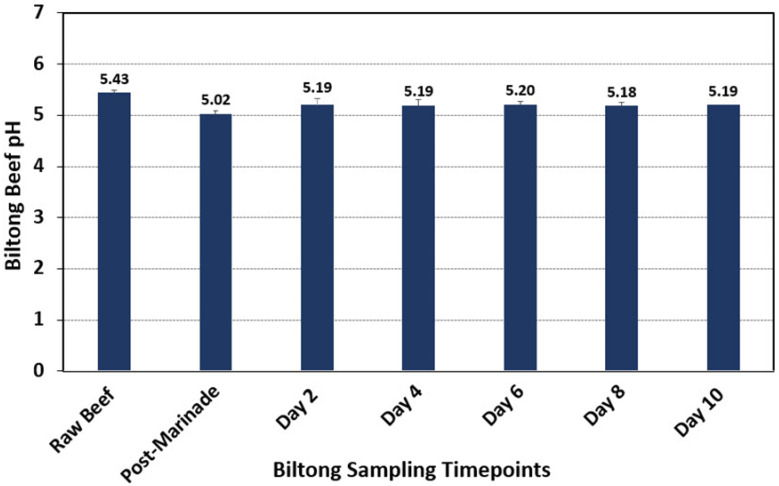
The pH of meat at each sampling timepoint during biltong processing. Samples were taken in triplicate at each timepoint following blending with sterile water in a laboratory blender (*n* = 6).

The initial pH of the raw meat pieces was approximately pH 5.43 (Figure 3), which was determined by blending beef samples in sterile water in a laboratory blender. The pH of the samples then decreased following the marination step to 5.02, which can be attributed to the presence of residual 100-grain red wine vinegar in the marinade. After removal from the marinade, the pH of biltong beef samples then equilibrated slightly higher to ~5.18–5.20 for the remainder of the drying process in the humidity-controlled oven (Figure 3). The pH of the marination solution was much lower (pH 2.5–2.7); during 30 min vacuum tumbling, the surface bacteria were immersed in the low-pH marinade solution, which could lead to cell death and inactivation of pathogenic bacteria [35,36], as observed in the current study and prior biltong trials where levels of inoculated pathogens were reduced after marination [4]. After removal from the vacuum tumbler, the residual marinade on the surface was absorbed, and the pH of biltong beef samples equilibrated to ~5.18–5.20 for the remainder of the drying process in the humidity oven (Figure 3).

#### 3.1.3. Temperature and Relative Humidity during Biltong Processing

**Figure 4:**
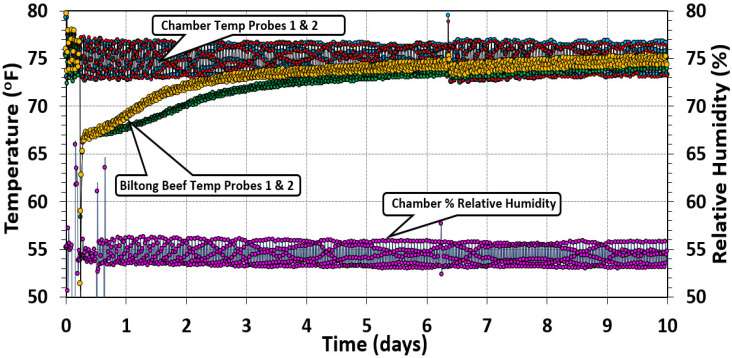
Oven temperature and relative humidity measurements. The temperature was set to 24.9 °C (75 °F), and the relative humidity setpoint was 55% RH during the drying process over a period of 10 days. Graphical data show the typical cycling of oven control above/below setpoint. Two temperature probes were placed in various places in the chamber and two additional probes were inserted into separate pieces of beef to track the internal temperature of the biltong product over the same drying period.

Temperature and RH measurements were recorded by computer software connected to the handheld temperature and humidity recorders to which the probes in the oven chamber were connected (Figure 4). Two temperature probes were inserted separately into two beef pieces to measure the internal beef temperature during processing, while the humidity probe was placed midway within the chamber. Air temperature and humidity were set to 23.9 °C (75 °F) and 55% throughout the duration of each trial but cycled above and below the set points. The internal temperature of the beef was more consistent and steadily increased from their initial temperature to match the temperature of the chamber. Long-term storage at low RH helps to evaporate moisture from the beef.

### 3.2. Surrogate Log Reductions during Biltong Processing

**Figure 5:**
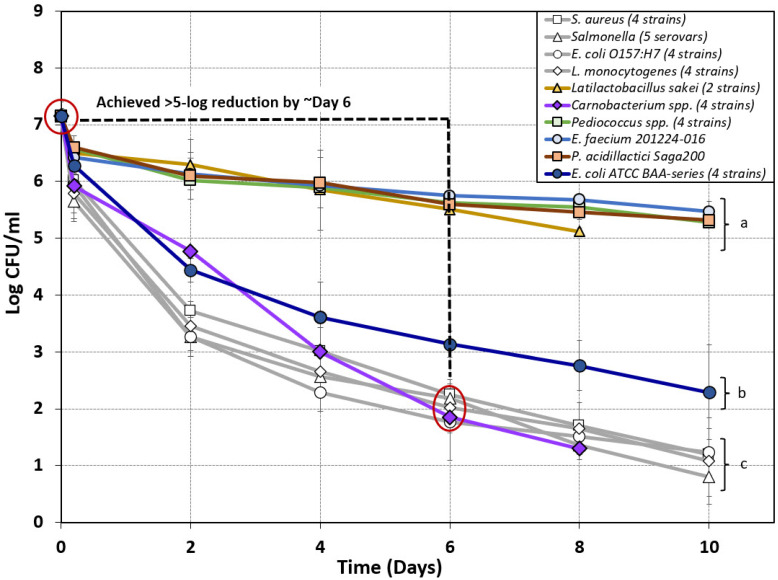
Composite graph of biltong processing data of nonpathogenic bacteria attempting to mimic the biltong process log reduction of pathogenic bacteria (light-gray lines) to be considered a possible ‘biltong processing surrogate’ organism for in-plant validation. Log reduction curves of various lactic acid bacteria (*Carnobacterium* spp., *Pediococcus* spp., *L. sakei*, and *E. faecium*) and Biotype I *E. coli* strains tested as potential surrogate organisms for biltong processing over a period of 8–10 days. Strains were compared to the log reduction curves observed during previous biltong validation studies using pathogenic bacteria including *Salmonella* serovars [4], *S. aureus, E. coli* O157:H7, and *L. monocytogenes* [5]. Data points are the mean of duplicate trials sampled in triplicate (*n* = 6). Statistical analysis was performed using one-way repeated-measures analysis of variance (RM-ANOVA) of the entire time course of data; curves with the same letter are not significantly different (*p* > 0.05); isolates with different letters are significantly different (*p* < 0.05).

Various bacteria were considered for examination as possible nonpathogenic surrogates, including strains recovered from biltong after processing. These included a two-strain mixture of *L. sakei* GO-R2C and GO-R2D and a four-strain mixture of *C. divergens* GO-R2E-B and GO-R1B and *C. gallinarum* NB-R2A and NB-R2B (Figure 5). We also examined a four-strain mixture of *P. acidilactici* and *P. pentosaceous* strains (*P. acidilactici* ATCC 8042 and PO2K5; *P. pentosaceous* ATCC 43200 and FBB61-2) vs. starter cultures that were available through culture companies (*E. faecium* 201224-016 and *P. acidilactici* Saga200) as surrogate organisms (Figure 5).

Only a slight reduction from inoculated levels was observed following vinegar/spice/salt marination (0.65, 0.58, 0.75, and 0.61 log reduction) with all cultures used, except for the four-strain mixtures of *Carnobacterium* spp. and *E. coli* ATCC BAA series (Figure 5). A larger log reduction was observed after marination of the four-strain mixtures of *Carnobacterium* spp. (1.23 log) and *E. coli* ATCC BAA-strains (0.86 log) (Figure 5). Trials using *E. coli* ATCC BAA (four-strain mix), *L. sakei* (two-strain mix)*, Pediococcus* spp. (four-strain mix), *E. faecium* 201224-016, and *P. acidilactici* Saga200 failed to achieve a 5 log reduction during biltong processing with overall reductions of 4.86 log, 2.03 log, 1.87 log, 1.68 log, and 1.83 log respectively. Of all the nonpathogenic strains examined, only the four-strain mixture of *Carnobacterium* spp. achieved an overall reduction of greater than 5 log (5.85 log) during the 8 day drying period (Figure 5). On the basis of these results, *Carnobacterium* spp. were the only organisms that achieved a 5 log reduction (within 6–8 days) comparable to that observed for the pathogenic strains, and they presented the best case for use as a *Salmonella, L. monocytogenes, E. coli* O157:H7, or *S. aureus* surrogate for biltong processing (Figure 5).

### 3.3. Comparison of Lyophilized/Frozen Starter Cultures with Metabolically Active (Grown) Versions in Biltong Processing Trials

**Figure 6:**
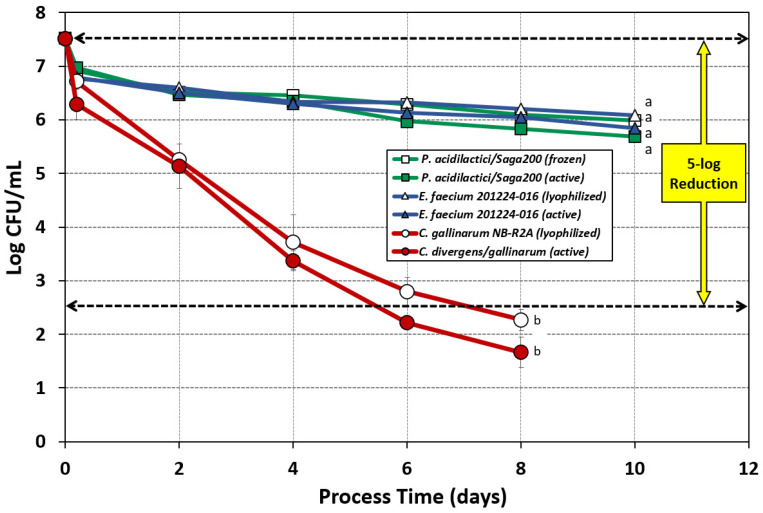
Biltong processing of beef inoculated with lyophilized/frozen cells vs. metabolically active cells (freshly grown) of *E. faecium* 201224-016, *P. acidilactici*, and *C. gallinarum* NB-R2A. Lyophilized *C. gallinarum* NB-R2A was compared to a four-strain cocktail of metabolically active *C. divergens/gallinarum*. Graph curves of frozen or lyophilized cultures have hollow symbols. Statistical analysis was performed using one-way repeated-measures analysis of variance (RM-ANOVA) over the entire time course of the datasets; graphs with the same letter are not significantly different (*p* > 0.05); isolates with different letters are significantly different (*p* < 0.05).

Several reports in the literature have used freeze-dried or frozen cultures, resuspended directly in buffer, to inoculate food samples in process trials for direct comparison to pathogens grown in microbiological media (which we describe as ‘active cultures’) [15,17]. The ease of availability of freeze-dried/frozen cultures from culture companies would facilitate the use of such cultures for in-plant validation studies; however, we were interested to see if they could provide the same response in a biltong process as the actively grown cultures (Figure 6). The comparisons were between two commercially available starter cultures, *E. faecium* 201224-016 (Vivolac Cultures; freeze-dried) and *P. acidilactici* Saga200 (Kerry Foods; frozen), and a lyophilized *C. divergens* NB R2A, which was chosen from among the *Carnobacterium* mixed strains demonstrating >5 log reduction in Figure 5.

Neither the lyophilized version of *E. faecium* 201224-016 (1.43 log reduction) nor the frozen version of *P. acidilactici* Saga200 (1.54 log reduction) achieved the 5 log reduction target; survival curves of the lyophilized/frozen forms were also not significantly different when compared to their metabolically active forms, i.e., 1.68 and 1.83 log reduction, respectively (Figure 6). The lyophilized single strain *C. divergens* NB R2A also showed no significant difference from the metabolically active culture and again achieved 5 log reduction during the biltong process (Figure 6). The data show that lyophilized or frozen versions of *E. faecium*, *P. acidilactici*, or *C. gallinarum* do not respond differently than actively grown cultures to biltong processing conditions and, when possible, their use might facilitate inoculated studies.

## 4. Conclusions

The lethality observed in the biltong process with *Carnobacterium* spp. aligned with that observed with four major pathogenic organisms indicating that *Carnobacterium* spp. could be an effective in-plant surrogate organism to monitor the effectiveness of biltong processing within a manufacturer’s facility. *Enterococcus faecium, L. sakei*, and *Pediococcus* spp. were not reduced much (<2 log) and were resilient toward the acid, salt, and low A_w_ experienced during 10 days of biltong processing. The use of lyophilized/frozen cells as inoculum for validation of biltong processing was not significantly different than using actively grown cells. This work helps to fill USDA-FSIS knowledge gaps in air-dried shelf-stable dried beef (biltong) processing with regard to potential surrogate organisms and critical factors involved in the biltong process. Future studies on biltong processing may include whether pathogens such as *Salmonella*, known to survive long periods of low water activity, can survive the extended shelf-life of biltong products to ensure that this does not become a possible (overlooked) problem.

## Figures and Tables

**Table 1:** List of strains used as challenge organisms for biltong processing in this study.

Organism	Strain Designation	Culture Collection Designation	Antibiotic Resistance (μg/mL) *	Source
*Pediococcus acidilactici*	ATCC 8042	PMM 128	GM, 10; RF, 5	Muriana Culture Collection
*Pediococcus acidilactici*	PO2K5	PMM 331	GM, 10; RF, 5	Muriana Culture Collection
*Pediococcus pentosaceous*	ATCC 43200	PMM 104	GM, 10; RF, 5	Muriana Culture Collection
*Pediococcus pentosaceous*	FBB61-2	PMM 105	GM, 10; RF, 5	Muriana Culture Collection
*Pediococcus acidilactici*	Saga200	PMM 444	NA, 10; CL, 10	Kerry Foods, Beloit, WI, USA
*Enterococcus faecium*	201224-016	PMM 445	NA, 10; CL, 10	Vivolac Cultures, Indianapolis, IN, USA
*Escherichia coli*	ATCC BAA-1427	PMM 876	OX, 1; NB, 2.5	ATCC, Muriana Culture Collection
*Escherichia coli*	ATCC BAA-1428	PMM 877	OX, 1; NB, 2.5	ATCC, Muriana Culture Collection
*Escherichia coli*	ATCC BAA-1429	PMM 878	OX, 1; NB, 2.5	ATCC, Muriana Culture Collection
*Escherichia coli*	ATCC BAA-1430	PMM 879	OX, 1; NB, 2.5	ATCC, Muriana Culture Collection
*Latilactobacillus sakei*	GO-R2C	PMM 446	GM, 2.5; RF, 2.5	Isolated from biltong
*Latilactobacillus sakei*	GO-R2D	PMM 447	GM, 2.5; RF, 2.5	Isolated from biltong
*Carnobacterium divergens*	GO-R2E-B	PMM 448	GM, 2.5; RF, 2.5	Isolated from biltong
*Carnobacterium divergens*	GO-R1B	PMM 449	GM, 2.5; RF, 2.5	Isolated from biltong
*Carnobacterium gallinarum*	NB-R2A	PMM 450	GM, 2.5; RF, 2.5	Isolated from biltong
*Carnobacterium gallinarum*	NB-R2B	PMM 451	GM, 2.5; RF, 2.5	Isolated from biltong

* Antibiotic designations: gentamicin, GM; rifamycin, RF; nalidixic acid, NA; colistin, CL; oxacillin, OX; novobiocin, NB.

## Data Availability

Not applicable.
